# Interferon regulatory factor 7 in inflammation, cancer and infection

**DOI:** 10.3389/fimmu.2023.1190841

**Published:** 2023-05-12

**Authors:** Furong Qing, Zhiping Liu

**Affiliations:** School of Basic Medicine, Gannan Medical University, Ganzhou, China

**Keywords:** IRF7, interferon, inflammation, cancer, infection

## Abstract

Interferon regulatory factor 7 (IRF7), a member of the interferon regulatory factors (IRFs) family, is located downstream of the pattern recognition receptors (PRRs)-mediated signaling pathway and is essential for the production of type I interferon (IFN-I). Activation of IRF7 inhibits various viral and bacterial infections and suppresses the growth and metastasis of some cancers, but it may also affect the tumor microenvironment and promote the development of other cancers. Here, we summarize recent advances in the role of IRF7 as a multifunctional transcription factor in inflammation, cancer and infection by regulating IFN-I production or IFN-I-independent signaling pathways.

## Introduction

Interferon regulatory factors (IRFs) are a class of transcription factor families that share a conserved N-terminal DNA-binding domain (DBD) and an IRF-associated structural domain (IAD) (1, 2). Currently, there are 11 members of IRFs. The mammalian IRFs consist of IRF1-9 (3), whereas IRF10 and IRF11 are only found in avian or fish (4, 5).The main role of IRFs is to regulate the transcription of Interferon (IFN) and the expression of IFN stimulated genes (ISGs). Thereby they promote the production of inflammatory cytokines and chemokine (6). Specifically, when this family member is activated by phosphorylation, it can further activate IFN and the expression of ISGs through the JAK-STAT pathway, and promote the production of inflammatory cytokines, which are widely involved in apoptosis, tumorigenesis and viral latency (7–9). In addition, the IAD structural domain at the C-terminus of IRFs can interact with other members of the family and other transcription factors including NF-κB and PU.1 to play important roles in host defense against viruses and bacteria, in innate and acquired immune responses, and in cell development and tumorigenesis (10).

Interferon regulatory factor 7 (IRF7) is a member of the IRFs. *IRF7* gene is located on human chromosome 11p15.5 and encodes four isoforms of IRF7A, -B, -C and -D (-H) (11), which can be constitutively expressed in the spleen, lymph nodes and bone marrow and especially in epithelial cells, monocytes and macrophages. Recognition of pathogenic microorganisms by pattern recognition receptors (PRRs) induces the activation and translocation of IRF7 to nuclear, leading to IFN-I production and secretion. Then IFN-I can be recognized and bound by IFNAR receptors on the cell surface. Subsequently, IRF7 production is induced via the JAK-STAT signaling pathway. This results in an IRF7-IFN-I positive feedback loop that allows for the sustained production of large amounts of IFN. However, many studies have shown that there are both promoting and inhibiting effects of IFN in diseases such as inflammation, cancer, and infection (12, 13). Therefore, by understanding the role of IRF7 in inflammation, cancer and infection, we can develop the new means to better regulate the function of IRF7 and modulate the immune response in a more precise and effective manner. More importantly, it will enable the development of highly targeted therapies for clinical use. However, systemic review of IRF7 in inflammation, cancer and infection is still lacking.

## IRF7 signaling

**Figure 1:**
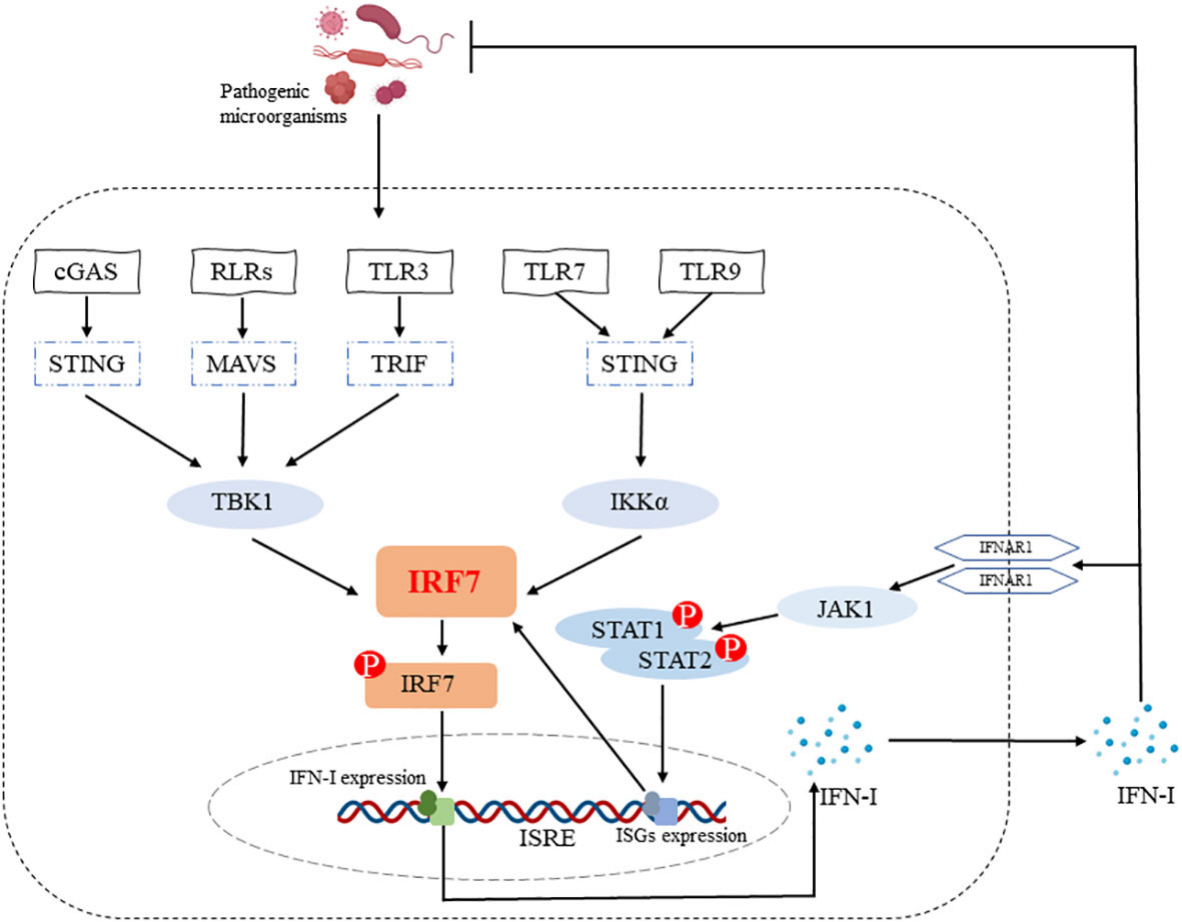
IRF7 promotes IFN-I production. IRF7 can be activated by phosphorylation through cGAS-STING-TBK1, RLRs-MAVS-TBK1, TLR3-TRIF-TBK1 and TLR7/TLR9-STING-IKKα signaling pathways. The entry of p-IRF7 into the nucleus induces IFN-I production and secretion, which plays a role in host defense against pathogenic microorganisms. In addition, IFN-I binds to IFNAR1 on the cell surface and subsequently induces the production of ISGs (e.g. IRF7) through the JAK1-STAT1/2 signaling pathway, ultimately forming an IRF7-IFN-I positive feedback loop.

The *IRF7* gene, originally identified in 1997 during latent EB virus (EBV) infection, encodes a protein that binds to and regulates the EB-Barr virus nuclear antigen 1 (EBNA-1) Q promoter (14) and is closely related to the major EBV oncogenic protein latent membrane protein-1 (LMP-1) (15). It has been shown that IRF7 can be activated by phosphorylation through TBK1/IKKϵ and TRIF-dependent pathways downstream of the cytoplasmic RNA/DNA sensor, and subsequently enters the nucleus, dimerizes with IRF3, exerts transcriptional activation and induces IFN-α/β expression (16). IRF7 is also essential for the induction of IFN-α/β gene expression via the viral-activated MyD88 independent pathway and the TLR-activated MyD88-dependent pathway (16). IRF7 is a positive regulator of *IFN-I* gene induction downstream of PRRs (17) and the positive feedback loop formed by IRF7-IFN-I allows for the continuous production of large amounts of IFN, which ultimately resists infection by pathogenic microorganisms (**Figure 1**).

## Role of IRF7 in inflammation

Inflammation is a host defensive response to detrimental stimuli. Usually, inflammation is an automatic and beneficial defense response. However, overt inflammation can be harmful, such as attacking on the body’s own tissues. Large amounts of cytokines, chemokines during inflammation could play important roles in the development of various diseases.

IRF7 can promote inflammation and the development of inflammatory diseases. Systemic sclerosis (SSc) is a multi-system autoimmune disease characterized by vasculopathy, fibrosis and immune system dysregulation. However, there are currently no targeted treatment options for the fibrotic complications of SSc, and disease-related mortality remains high (18). A study showed that the gene and protein levels of IRF7 were significantly enhanced in skin and cultured fibroblasts from patients with SSc. Furthermore, IRF7 promotes TGF-β-induced fibrosis by interacting with Smad3 in fibroblasts. Specifically, in comparison with wild-type (WT) mice, IRF7 knockout (*Irf7^-/-^*) mice showed lower *IL-6* gene expression levels, lower expression of pro-fibrotic factors in fibroblasts, less subcutaneous thickness, and milder inflammatory responses, and thus less skin fibrosis after bleomycin stimulation (19). Both type 2 helper T (Th2) cells and type 2 innate lymphoid cells (ILC2s) are major driver of allergic airway inflammation or asthma. Patients with asthma displayed higher levels of ILC2s in both peripheral blood and bronchoalveolar lavage fluid (BALF) compared to healthy individuals (20). Another study showed that IRF7 expression in murine lung ILC2s was dramatically induced upon stimulation of papain or interleukin-33 (IL-33). ILC2s from asthma patients display a markedly higher level of IRF7 than those from healthy donors, indicating that IRF7 might enhance the development of asthma. Furthermore, IRF7 deficiency in mice attenuated the various allergic asthma animal models through limiting the expansion and function of lung ILC2s by inhibiting the expression of BCL11B and other cytokines such as IL-13 and IL-5 (21). Similarly, IRF7, together with IRF3, was shown to promote RAPTOR and mTOR activation and inhibit autophagy, which in turn enhanced lung inflammation and injury induced by diesel exhaust particles (DEPs) (22).

However, IRF7 is also able to suppress inflammation and inhibit the development of inflammatory diseases in other circumstances. Pulmonary hypertension (PH) is a severe syndrome characterized by the extensive remodeling of small intrapulmonary arteries, leading to the development of right ventricular hypertrophy and dysfunction and, eventually, lethal right heart failure (23). Current treatment options primarily improve the symptoms or slow disease progression, but they cannot cure this severe condition. Thus, the development of more effective treatments is urgently warranted (24). Using an MCT-induced *in vivo* rat model of PH and *in vitro* rat pulmonary artery smooth muscle cells (PASMCs), IRF7 was able to inhibit NF-κB activation resulting in a significant reduction in the levels of pro-inflammatory cytokines such as TNF-α and IL-6, as well as inhibit ATF3 signaling to reduce the proliferation of PASMCs and their resistance to apoptosis. These results suggest that IRF7 can prevent vascular remodeling in pulmonary arterial hypertension by inhibiting the proliferation and inflammation of PASMCs (25).

**Figure 2:**
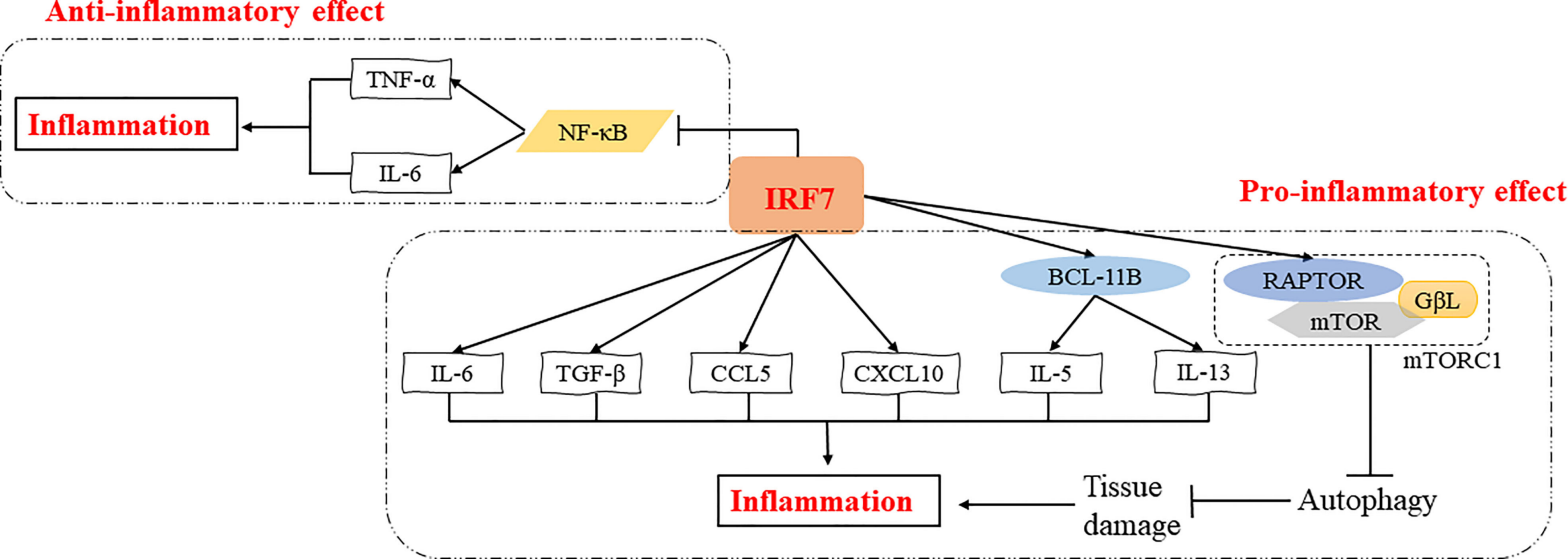
Regulatory mechanisms of IRF7 in the inflammatory response. IRF7 can exert pro-inflammatory effects by promoting the expression of IL-5 and IL-13 induced by BCL11B or directly promoting the expression of inflammatory cytokines and chemokines such as IL-6, TGF-β, CCL5 and CXCL10. IRF7 can also induce the expression of RAPTOR, which in turn activates mTORC1 thus inhibiting autophagy and promoting inflammation by increasing tissue damage. In addition, IRF7 can suppress NF-κB signaling pathway and inhibit the expression of TNF-α and IL-6, exerting an anti-inflammatory effect.

Overall, transcription factors are key factors that regulate the inflammatory response (26). Notably, IRF7 is a multifunctional transcription factor, and plays pro-inflammatory or anti-inflammatory roles in different inflammatory diseases through various signaling pathways, such as NF-κB signaling pathway, autophagy activation, etc. (**Figure 2**). The specific role of IRF7 in inflammation may vary depending on the microenvironment (**Table 1**).

**Table 1:** The role of IRF7 in inflammation, cancer and infection.

Category	Diseases	Cells	Research Methods	Mechanism	Function	Reference
Inflammation	Systemic Sclerosis	Fibroblast	Bleomycin-induced skin fibrosis modeling in WT and *Irf7^-/-^* mice; SSc patient samples.	IRF7 promoted IL-6 expression and inflammatory response and interacted with SMAD3 in fibroblasts to enhance TGF-β-induced fibrosis.	Promotion	*Wu M, Ann Rheum Dis, 2019* (19)
	Bronchial asthma	Type 2 innate lymphoid cells	*In vivo* allergic asthma modeling was performed on WT and *Irf7^-/-^* mice, while ILC2s cells from WT and *Irf7^-/-^* mice were cultured *in vitro* and subsequently studied in combination with samples from asthmatic patients.	In an allergic asthma model, IRF7 mediated the activation and function of ILC2s cells through BCL11B, increasing the expression of IL-5 and IL-13, which in turn promoted allergic respiratory inflammation and allergic asthma.	Promotion	*Juan He, Cell Rep, 2019* (21)
	Acute pneumonia	Macrophages	Modeling of acute lung inflammation induced by DEPs and *in vitro* BMDM, BMDC and neutrophil cultures in WT and *Irf3^-/-^Irf7^-/-^* mice.	In a model of acute lung inflammation induced by diesel exhaust particles (DEPs), IRF7 was able to induce RAPTOR expression together with IRF3, promote mTORC1 activation and signal transduction, inhibit autophagy, and thus promoting lung injury induced by DEPs.	Promotion	*Yang Li, Eur J Immunol, 2020* (22)
	Pulmonary hypertension	Pulmonary artery smooth muscle cells	MCT-induced pulmonary hypertension was studied in rats and rat PASMCs cells transfected with an adenovirus overexpressing IRF7.	IRF7 was able to inhibit MCT-induced NF-κB activation in PH rats and large PASMCs cells, causing a significant decrease in the levels of pro-inflammatory cytokines such as TNF-α and IL-6, while inhibiting ATF3 signaling to reduce the proliferation of PASMCs and promoting apoptosis.	Inhibition	*Deng Y, Life Sci, 2021 (* 25)
Cancer	Prostate cancer	NK cells	*In vitro* culture of prostate cancer cells stably transfected with IRF7 and *in vivo* experiments with nude mice, as well as validation with clinical patient samples.	Overexpression of IRF7 increased IFN-β production and thus significantly enhanced NK cell activity, leading to cytolysis of prostate cancer target cells and exerting an inhibitory effect on bone metastasis of prostate cancer.	Inhibition	*Zhao Y, Oncol Res, 2017 (* 27)
	Gastric carcinoma	Gastric cancer cells	*In vitro* culture of gastric cancer cell lines and *in vivo* tumor growth assays via nude mice.	The circ0007360/miR-762/IRF7 axis inhibited the survival, migration and invasion of gastric cancer cells and slowed down the progression of gastric cancer.	Inhibition	*Xing Y, Front Cell Dev Biol, 2022 (* 28)
	Breast cancer	Breast cancer cells, Macrophages	Clinical breast cancer patient samples and *in vitro* cultured breast cancer cells.	IRF7 that is regulated by miR-762 could inhibit the proliferation and invasion of breast cancer cells.	Inhibition	*Li Y, Cell Prolif, 2015 (* 29)
	Lewis Lung Cancer	Bone marrow progenitor cells	Tumor growth assays and *in vitro* culture tumor cell studies on WT and *Irf7^-/-^* mice. Clinical patient samples.	IRF7 inhibited the expression of S100A9, and inhibited the aggregation of granulocyte-like myeloid suppressor cells (G-MDSCs), thus suppressing tumor metastasis.	Inhibition	*Yang Q, Oncogene, 2017 (* 30)
	Glioblastoma multiforme	Microglia, Macrophages	*In vitro* culture of glioma cells transfected with overexpressed/silenced IRF7 plasmids and *in vivo* study of nude mice injected with tumor cells.	①IRF7 inhibited the expression, biosynthesis and function of argonaute 2 (AGO2), a regulator of microRNA maturation, thus promoting glioma cell invasion as well as chemo- and radio-resistance. ②IRF7 upregulated the expression of anti-inflammatory genes such as *IL10*, downregulated pro-inflammatory genes such as *IL1B*, *TNF*, *CXCL1* and *CXCL2*, and polarized microglia to the M2 type, thereby establishing a tumor microenvironment that exerted immunosuppressive effects. Meanwhile, IRF7 mediated STAT3 expression and promoted the survival and stem cell differentiation potential of glioblastoma multiforme (GBM) cells through the IL-6-STAT3 signaling pathway, thus enhancing the progression and invasion of GBM.	Promotion	*Kim JK, Tumour Biol, 2015 (* 31) *Tanaka T, Glia, 2015 (* 32) *Li Z, J Cancer, 2017 (* 33) *Cohen M, EMBO J, 2014 (* 34)
	Breast cancer	Macrophages	Tumor growth *in vivo* by injecting macrophages and breast cancer cells to nude mice.	IRF7 promoted the polarization of tumor-associated macrophages (TAMs) to M2 type, inhibited anti-inflammatory factor production, enhanced immune escape of tumor cells, proliferation and migration of breast cancer cells, and thus promoting the development of breast cancer.	Promotion	*Tu D, Cell Biol Int, 2021 (* 35)
	Non-small cell lung cancer	Non-small cell lung cancer cells	Clinical patient samples and *in vitro* cultured NSCLC cells were studied.	IRF7 activated a positive feedback regulatory loop of interferon signaling and subsequently activated the RIG-I-like receptor signaling pathway and BCL-2 expression to inhibit apoptosis and promote proliferation, invasion and migration of non-small cell lung cancer cells.	Promotion	*Tang XD, Cell Physiol Biochem, 2018 (* 36)
	Renal cell carcinoma	Renal cancer cells	Clinical patient samples and *in vitro* cultured kidney cancer cells.	IRF7 enhanced the proliferation and invasion of kidney cancer cells.	Promotion	*Lin L, J Cell Biochem, 2020 (* 37)
Bacterial infection	*Pseudomonas aeruginosa*	Dendritic cells	*In vivo* infection of WT and *PTP1B^-/-^* mice and *in vitro* culture of mouse bone marrow-derived dendritic cells.	IRF7 promoted the activation of interferon-stimulated response element (ISRE) and its downstream cytokine and chemokine production, such as CCL5, CXCL10 and IFN-β, which in turn inhibited *P. aeruginosa* infection.	Inhibition	*Yue L, Am J Pathol, 2016 (* 38)
	*Mycobacterium tuberculosis*	Macrophages	Mouse bone marrow-derived macrophages and human-derived macrophage cell lines *in vitro*.	IRF7 can be activated by weakly virulent *Mycobacterium tuberculosis*, acting synergistically with IRF3 to increase IFN-I production and thus control infection. IRF7 can be inhibited by OASL, a negative regulator of IFN-I activated by strongly virulent *Mycobacterium tuberculosis*, to suppress inhibition and produce a cytokine storm that ultimately leads to macrophage death.	Inhibition	*Leisching G, Virulence, 2017 (* 39)
	*Mycobacterium*	Macrophages	①*In vivo* infection of WT, *Irf7^-/-^*, *Irf3^-/-^*, *cGAS^-/-^*, *STING^-/-^* mice and *in vitro* culture of RAW264.7 cells. ②*In vivo* infection of WT and *Mavs^-/-^* mice and *in vitro* culture of BMDM.	IRF7 promoted IFN-β expression and inhibited *Mycobacterium* infection.	Inhibition	*Ruangkiattikul N, Virulence, 2017 (* 40) *Cheng Y, J Exp Med, 2018 (* 41)
Parasitic infection	*Cryptosporidium parvum*	Epithelial cells	Culturing intestinal epithelial cell lines and mouse intestinal tissues *in vitro* and infecting mice *in vivo*.	IRF7 promoted IFN-I expression and NF-κB signaling pathway, which in turn promoted intestinal epithelial resistance to *Cryptosporidium parvum* infection.	Inhibition	*Mathy NW, Front Immunol, 2022 (* 42)
	*Plasmodium*	Dendritic cells, Macrophages	*In vivo* infection of WT, *Irf7^-/-^* and *Ifnar1^-/-^* mice.	IRF7 promoted IFN-α isoform production, inhibited CD4^+^ Th1 cell activation, and suppressed Th1 responses in the spleen, thereby promoting *Plasmodium* infection.	Promotion	*Edwards CL, Eur J Immunol, 2015 (* 43)
Viral infection	Venezuelan equine encephalitis virus (RNA virus)	Macrophages	*In vivo* infection of WT and *Irf7^-/-^* mice and *in vitro* culture of BMDM, BMDC, and macrophage cell lines.	IRF7 promoted the production of IFN-I and inhibited VEEV infection.	Inhibition	*Bhalla N, J Virol, 2019 (* 44)
	West Nile virus (RNA virus)	Macrophages, Dendritic cells, Fibroblasts	*In vivo* infection of WT and *Irf7^-/-^* mice and *in vitro* culture of BMDM, BMDC, MEF, and primary cortical neurons.	IRF7 promoted IFN-α gene expression and protein production in macrophages, fibroblasts, dendritic cells and cortical neurons, thereby suppressing WNV infection in the peripheral and central nervous system.	Inhibition	*Daffis S, J Virol, 2008 (* 45)
	Rhinovirus (RNA virus)	Macrophages, Neutrophils, Eosinophils	*In vivo* infection studies were performed by injecting mice with anti-CCL7 antibody/isotype control, IRF7-siRNA/control siRNA.	IRF7 promoted IFN-α and IFN-β production, as well as neutrophil and macrophage infiltration in combination with CCL7, thus enhancing RV-induced antiviral immune responses.	Inhibition	*Girkin J, J Immunol, 2015 (* 46)
	Influenza A virus (RNA virus)	/	*In vivo* infection of WT and *p53^-/-^* mice.	The activation of IRF7 through p53 signaling promoted IFN-γ production, while antiviral signals such as MX2 and EIF2AK2 were enhanced, thereby preventing immune escape from influenza A virus.	Inhibition	*Yan W, BMC Med Genomics, 2015 (* 47)
	Human immunodeficiency virus (RNA virus)	Monocytes/Macrophages	*In vitro* culture of human cervical tissue and peripheral blood single nucleated cells for infection studies.	IRF7 promoted the production of IFN-α and downregulated RELA expression, a member of the NF-κB family, thereby inhibiting HIV replication in cervical tissue.	Inhibition	*Rollenhagen C, PLoS One, 2015 (* 48)
	SARS-CoV-2 (RNA virus)	Neutrophils, Plasmacytoid dendritic cells, Fibroblasts	Analysis with patient blood specimens.	①IRF7 promoted the production of inflammatory cytokines by neutrophils, leading to the development of an inflammatory cytokine storm, which enhanced SARS-CoV-2 infection. ②IRF7 promoted the production of IFN-I and IFN-III, which inhibited SARS-CoV-2 infection.	Promotion/Inhibition	*Ai Z, PLoS One,2022 (* 49) *Campbell TM, J Exp Med,2022 (* 50)
	Herpes C virus (DNA virus)	Macrophages	*In vivo* infection of WT and *Irf7^-/-^* mice, and *in vitro* culture of BMDM.	IRF-7 attenuated chronic viral infection by limiting the latency and establishment of viral reactivation of herpes C virus in the peritoneal cavity and, to a lesser extent, in the spleen.	Inhibition	*Johnson KE, J Virol, 2020 (* 51)

## Role of IRF7 in cancer

As a multifunctional transcription factor, IRF7 can regulate cell differentiation, proliferation, and apoptosis in addition to being involved in immune regulation. Therefore, IRF7 plays an important role in tumor development and metastasis (10).

IRF7 can act as a tumor suppressor that inhibits the proliferation and metastasis of cancer cells. A study showed that overexpression of IRF7 increased IFN-β production and significantly enhanced NK cell activity, leading to cytolysis of prostate cancer cells and exerting a role in limiting bone metastasis of prostate cancer (27). Another study on different gastric cancer cell lines revealed that the activation of circ0007360/miR-762/IRF7 axis suppressed the survival, migration and invasion of gastric cancer cells, indicating the inhibitory role of IRF7 in gastric cancer (28). A further study showed that miR-762 can directly target the 3′ UTR of IRF7 mRNA and inhibit IRF7 expression and in turn promotes the proliferation and invasion of breast cancer cells (29). Meanwhile, miR-1587 can downregulate IRF7 expression to promote the polarization of tumor-associated macrophages (TAMs) to M2 type, which produces anti-inflammatory factors to promote immune escape, proliferation and migration of breast cancer cells, and thus enhancing breast cancer development (35). IRF7 was also shown to inhibit the expansion of granulocytic myeloid-derived suppressor cells (G-MDSCs) through directly suppressing S100A9 expression, thus reducing lung cancer metastasis (30).

Interestingly, IRF7 can also act as an oncogene that promotes cancer development. IRF7 was shown to promote glioma cell invasion as well as chemoresistance and radiation resistance by inhibiting the expression of argonaute 2 (AGO2), a regulator of microRNA maturation, biosynthesis, and function (31). Another study showed that lncRNA AFAP1-AS1 promoted non-small cell lung cancer (NSCLC) cell proliferation, invasion and migration while inhibiting cell apoptosis. Meanwhile, lncRNA AFAP1-AS1 can activate IRF7, indicating that IRF7 can promote cell proliferation (36). A further study found that Circ-EGLN3 could act as an endogenous competitive RNA (ceRNA) to enhance the expression of IRF7 by competitively binding miR-1299, which in turn promoted the cell proliferation and invasion and the development of renal cell carcinoma (37). In addition, IRF7 can upregulate the expression of anti-inflammatory genes such as *IL10* and downregulate pro-inflammatory genes such as *IL1B*, *TNF*, *CXCL1* and *CXCL2* to polarize microglia to the M2 type, thereby establishing a tumor microenvironment that exerts immunosuppressive effects. Meanwhile, IRF7 mediates STAT3 expression and promotes the survival and stem cell differentiation potential of glioblastoma multiforme (GBM) cells through the IL-6-STAT3 signaling pathway, and then enhances the progression and invasion of GBM. Notably, IRF7 promotes the conversion of microglia to M2 type, a pathway that can be inhibited by TGFβ1 (32–34).

**Figure 3:**
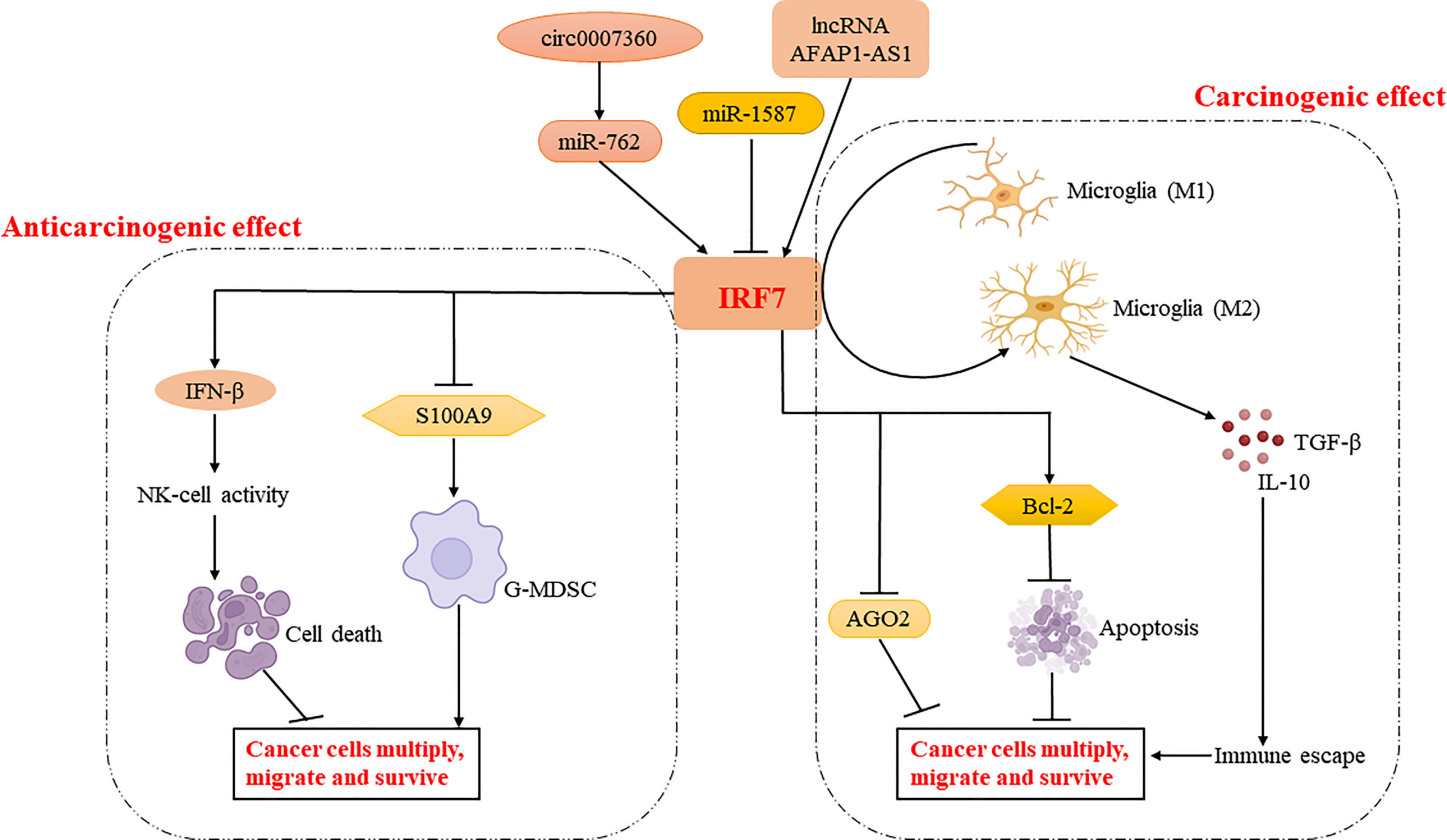
Regulatory mechanisms of IRF7 in cancer cell proliferation, migration and survival. IRF7 inhibits tumor development by enhancing NK cell activation via regulating IFN-β production and reducing the aggregation of G-MDSC via regulating S100A9 production. Meanwhile, IRF7 may enhance tumor development by regulating the expression of AGO2 and BCL-2, or promoting phenotypic shift of microglia to M2 type. Interestingly, IRF7 is regulated by circ0007360-miR-762/lncRNA AFAP1-AS1/miR-1587 and plays an important role in the tumor microenvironment.

As mentioned above, IRF7 plays different roles in different tumor progressions. It can act as a tumor suppressor to kill cancer cells by promoting NK cell activity or to function downstream of cancer cell miRNAs (27, 28). IRF7 can also downregulate the expression of S100A9 and reduce the aggregation of G-MDSCs to limit the metastasis and spread of cancer cells (30). In addition, it can restrict the polarization of tumor-associated macrophages to M2 macrophages, thus preventing the proliferation, migration and metastasis of cancer cells (35). IRF7 can also act as a pro-oncogene to inhibit the expression of microRNA regulators and apoptosis of cancer cells. Meanwhile, it can promote IL-6-STAT3 signaling to induce cancer cells to acquire stem cell differentiation potential (33). Moreover, it can regulate the phenotypic transition of microglia and enhance the expression of anti-inflammatory genes, thereby inducing cancer cell proliferation, invasion and immune escape (32) (**Figure 3**). Therefore, the role of IRF7 in cancer development may be different in various conditions (**Table 1**).

## Role of IRF7 in various infections

### Role of IRF7 in viral infections

Innate antiviral signaling mainly activates IRF3, IRF7 and NF-κB signaling to induce IFN and other pro-inflammatory factors to inhibit viral replication and propagation. *Irf7^-/-^* mice infected with Venezuelan equine encephalitis virus (VEEV) had significantly lower survival rates and higher viral loads in liver, spleen and brain tissues compared to WT mice due to reduced IFN-I production (44). Compared to WT mice, *Irf7^-/-^* mice exhibited higher lethality, less IFN-I production, and early and elevated viral loads in peripheral and central nervous system tissues after West Nile virus (WNV) infection. Consistent with this, there was reduced IFN-α production and increased viral titers in *Irf7^-/-^* progenitor macrophages, fibroblasts, dendritic cells, and cortical neurons (45). Another study showed that IRF7 silencing inhibited the production of IFN-α and IFN-β, while IRF7 could act in combination with CCL7 to increase neutrophil and macrophage infiltration and therefore enhancing antiviral response induced by rhinovirus RV infection (46).

Influenza A virus inhibited p53-IRF7-IFN-γ signaling, resulting in diminished expression of antiviral molecules such as MX2 and EIF2AK2, leading to immune escape (47). IRF7 promotes the production of IFN-α and is also able to downregulate the expression of RELA, a member of the NF-κB family, thereby inhibiting the replication of human immunodeficiency virus (HIV) in cervical tissue (48). Notably, IRF7 was shown to attenuate chronic infection by limiting the establishment of herpes C virus latency and viral reactivation in the peritoneal cavity and, to a lesser extent, in the spleen (51).

Some studies investigated the relation between IRF7 and SARS-CoV-2. Single-cell RNA-seq analysis of neutrophils from COVID-19 patients revealed that excessive activation of neutrophils in critically ill patients was significantly and positively correlated with the expression of IRF7. That is, activation of IRF7 promoted neutrophil production of inflammatory cytokines during SARS-CoV-2 infection, leading to the development of an inflammatory cytokine storm. IRF7 signaling blockade effectively reduced neutrophil inflammation during SARS-CoV-2 infection. Noteworthy, IRF7 expression was positively correlated with SARS-CoV-2 RNA load in virus-positive neutrophils (49). However, another study found that patients with autosomal recessive IRF7 deficiency had reduced type I and type III IFN production by respiratory epithelial cells and plasmacytoid dendritic cells, and were highly susceptible to SARS-CoV-2 infection (50).

Interestingly, IRF7 deficient mice were unable to control lymphocytic choriomeningitis virus (LCMV) replication due to reduced IFN-I production in the early stages of infection. However, in the late stages of infection, they did mount a normal CD4^+^ T cell response, a relatively normal CD8^+^ T cell response, enabling the clearance of LCMV infection to a degree consistent with that of WT mice (52). Another study also found that CD8^+^ T cell recruitment to the CNS and clearance of LCMV were IRF7-independent (53). These indicate that IRF7 may not play an intrinsic role in the activation of T cells. In short, IRF7 enhances the host defense against RNA viral infection by promoting IFN-I production in both acute and chronic phases.

However, some viruses are able to undergo immune escape by targeting IRF7. For example, Seneca Valley Virus (SVV) induced degradation of IRF3 and IRF7 proteins and inhibited transcription of IFN-α, IFN-β, and ISGs, thus leading to immune escape of the virus (54). African swine fever virus (ASFV) MGF505-7R inhibited IFN-β and ISRE promoter activity and the expression of IFN-I and ISGs by degrading IRF7 and TBK1, thereby evading the host antiviral response (55). VP23 protein from Marek’s disease virus (MDV) inhibited IRF7 phosphorylation and nuclear translocation, leading to reduced IFN-β production and ultimately immune escape (56). These also show that IRF7 is important in inducing a positive feedback loop in IFN and viral immune escape.

### Role of IRF7 in bacterial infections

IRF7 plays an important role in the host defense against bacterial infection by regulating IFN-I. A study found that protein tyrosine phosphatase-1B (PTP1B) deficiency could enhance ISRE and the production of cytokine and chemokine, thus increase host clearance of *Pseudomonas aeruginosa.* Meanwhile, it was found that PTP1B deficiency induce the expression of IRF7, indicating that IRF7 could increase host defense against *P. aeruginosa* infection by promoting the production of IFN-I (38). When weakly virulent *Mycobacterium tuberculosis* infects macrophages, it can activate IRF3 and IRF7, resulting in increased IFN-I production and thus controlling the infection, while strongly virulent *M. tuberculosis* inhibits IRF7 expression and produces cytokine storm due to its ability to activate Oasl1, a negative regulator of IFN-I, which ultimately leads to cell death of macrophage, indicating the inhibitory role of IRF7 in *M. tuberculosis* infection (39). Other studies also found that upregulation of IRF7 through the activation of cGAS-STING-TBK1-IRF3/IRF7 and RIG-I-MAVS-IRF7 signaling pathways can promote IFN-β expression and directly inhibits *M. tuberculosis* infection (40, 41).

### Role of IRF7 in parasitic infections

Given its multifunctional transcription factor activity, IRF7 also has an important role in host defense against parasitic infections. IRF7 was reported to promote IFN-I expression and enhances intestinal epithelial antimicrobial defense against *Cryptosporidium parvum* infection (42). Interestingly, IRF7 was shown to impair early splenic CD4^+^ Th1 cell activation, thereby promoting infection by blood-stage *Plasmodium (* 43). Therefore, the role of IRF7 in parasitic infections remains to be characterized.

**Figure 4:**
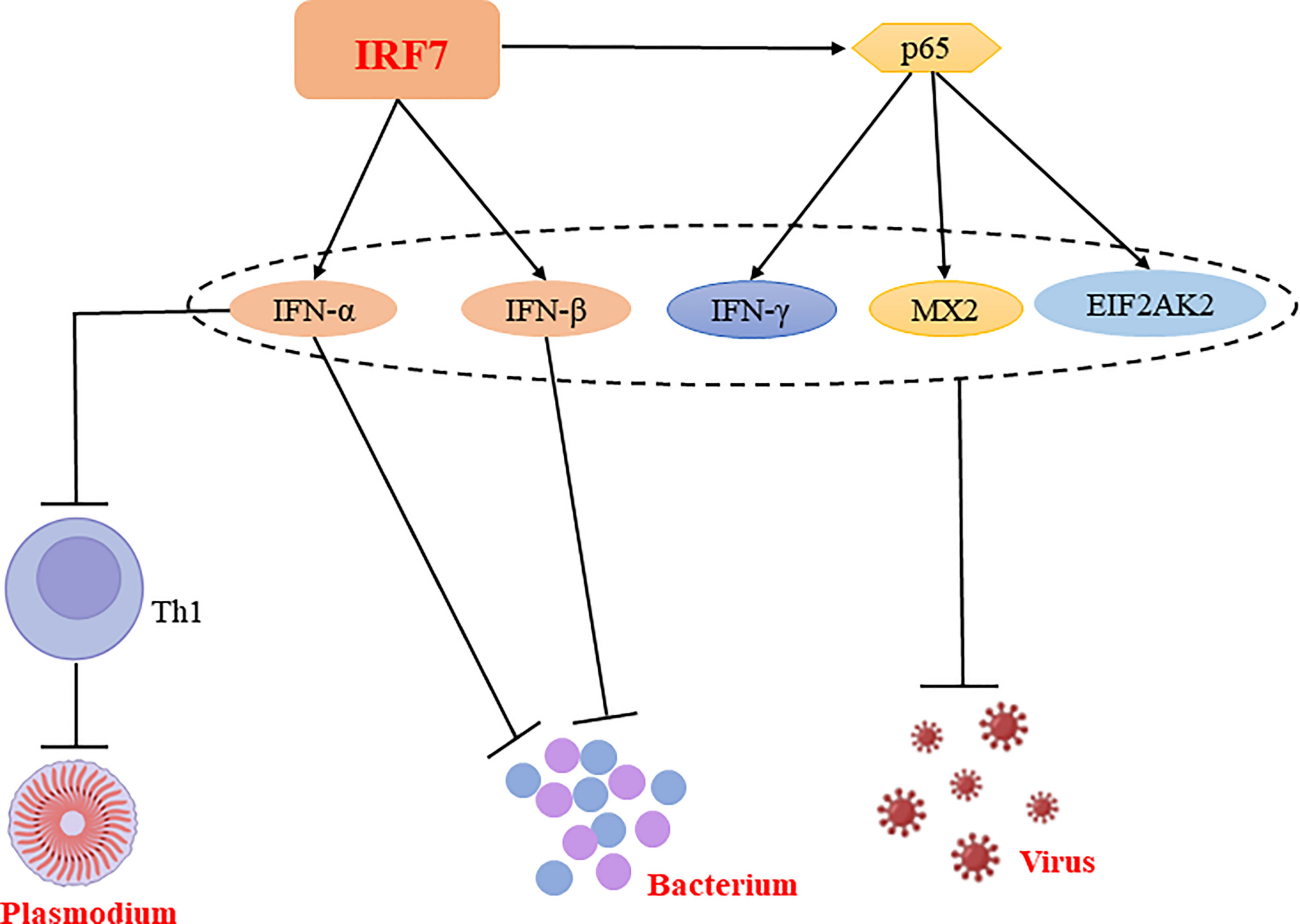
Regulatory mechanisms of IRF7 in bacterial, parasitic and viral infections. IRF7 is able to promote the production of IFN-α and IFN-β, thereby inhibiting bacterial replication and suppressing bacterial infections. Similarly, IRF7 is able to exert antiviral effects not only by promoting the expression of IFN-α and IFN-β; it also exerts antiviral effects by promoting the expression of p65, which enhance the production of antiviral molecules such as IFN-γ, MX2 and EIF2AK2. Notably, IFN-α is able to inhibit CD4^+^ Th cell activation and suppress Th1 responses, thereby promoting Plasmodium infection.

In conclusion, IRF7 plays a predominantly resistant role in bacterial and viral infections and has both a facilitative and inhibitory role in parasitic infections, which may be related to the different cells or signaling pathways (**Figure 4**, **Table 1**).

## Conclusion

A rapid, precise and ordered cellular response is central for host defense against pathogens and tumors. IRF7 plays a crucial role as an important transcriptional regulator of cellular responses in a variety of inflammatory diseases, cancers and infections. IRF7, a major regulator of IFN-I, exerts different biological functions and activities in various inflammation-related diseases. IRF7 can be either a tumor suppressor or an oncogene. As an interferon regulatory factor, IRF7 also plays a key role in host defense against bacterial, parasitic and viral infections. However, its role in fungal infections has not been well characterized.

It is undeniable that IRF7 plays a key role in host defense against various bacterial and viral infections by promoting the production of IFN-I. However, many studies have now proposed other functions for IRF7. As a transcription factor, IRF7 can directly induce the expression of inflammatory cytokines and autophagy by promoting mTOR signaling pathway. IRF7 also plays an important role in immune escape, cell proliferation and survival of tumor cells by affecting macrophage polarization, NK cell activation, apoptosis, and G-MDSC cell aggregation. Interestingly, IRF7 can be regulated by some MicroRNAs and also affects certain MicroRNA regulators in the tumor microenvironment. A study showed that, miR-541 promoted vascular smooth muscle cell proliferation by targeting IRF7 (57). In contrast, miR-144 was shown to target the TRAF6-IRF7 axis, whose activation attenuates the host response to influenza virus infection. This indicates that mechanisms to induce IRF7 activity by microRNAs, directly or indirectly, exist (58). In addition, IRF7 is subject to ubiquitination modifications during viral infection. TRIM21 affects IRF7 stability downstream of viral TLRs in order to limit antiviral responses (59). Like TRIM21, the E3 ligase RAUL adds K48 linked ubiquitin chains to both IRF3 and IRF7 and ultimately acts as a brake on the system in response to viral infection (60).Therefore, whether IRF7, as a transcription factor, promotes or inhibits disease development by directly or indirectly regulating additional molecules deserves further exploration; whether IRF7, as an interferon regulator, functions independently of IFN-I or through IFN-II or IFN-III in inflammation, cancer and infection remains unclear.

In conclusion, IRF7 is a multifunctional regulator that not only mediates the production of IFN-I, but also regulates autophagy, apoptosis and cell activation, and proliferation. Therefore, IRF7 plays different roles in various diseases, which may result from activation of multiple signaling pathways. It deserves more investigations to study the regulatory mechanisms of IRF7 and the signaling pathways of the IRF system, which may become important targets for the treatment of infectious diseases, inflammation-related diseases or cancer.

## Author contributions

Original idea and planning: ZL, Writing. FQ Reviewing and editing—ZL. All authors contributed to the article and approved the submitted version.

## References

[1] Chen W Royer WE Jr. Structural insights into interferon regulatory factor activation. Cell Signal (2010) 22(6):883–7. doi: 10.1016/j.cellsig.2009.12.005 PMC2846214 20043992

[2] Czerkies M Korwek Z Prus W Kochanczyk M Jaruszewicz-Blonska J Tudelska K. Cell fate in antiviral response arises in the crosstalk of IRF, NF-kappaB and JAK/STAT pathways. Nat Commun (2018) 9(1):493. doi: 10.1038/s41467-017-02640-8 29402958 PMC5799375

[3] Bin L Li X Richers B Streib JE Hu JW Taylor P. Ankyrin repeat domain 1 regulates innate immune responses against herpes simplex virus 1: a potential role in eczema herpeticum. J Allergy Clin Immunol (2018) 141(6):2085–93.e1. doi: 10.1016/j.jaci.2018.01.001 29371118 PMC5994174

[4] Nehyba J Hrdlickova R Burnside J Bose HR Jr. A novel interferon regulatory factor (IRF), IRF-10, has a unique role in immune defense and is induced by the v-rel oncoprotein. Mol Cell Biol (2002) 22(11):3942–57. doi: 10.1128/MCB.22.11.3942-3957.2002 PMC133824 11997525

[5] Suzuki Y Yasuike M Kondo H Aoki T Hirono I. Molecular cloning and expression analysis of interferon regulatory factor 10 (IRF10) in Japanese flounder, paralichthys olivaceus. Fish Shellfish Immunol (2011) 30(1):67–76. doi: 10.1016/j.fsi.2010.09.010 20883793

[6] Wang S Lin Y Yuan X Li F Guo L Wu B. REV-ERBalpha integrates colon clock with experimental colitis through regulation of NF-kappaB/NLRP3 axis. Nat Commun (2018) 9(1):4246. doi: 10.1038/s41467-018-06568-5 30315268 PMC6185905

[7] Tamura T Yanai H Savitsky D Taniguchi T. The IRF family transcription factors in immunity and oncogenesis. Annu Rev Immunol (2008) 26:535–84. doi: 10.1146/annurev.immunol.26.021607.090400 18303999

[8] Kumar M Garand M Al Khodor S. Integrating omics for a better understanding of inflammatory bowel disease: a step towards personalized medicine. J Transl Med (2019) 17(1):419. doi: 10.1186/s12967-019-02174-1 31836022 PMC6909475

[9] Honda K Taniguchi T. IRFs: master regulators of signalling by toll-like receptors and cytosolic pattern-recognition receptors. Nat Rev Immunol (2006) 6(9):644–58. doi: 10.1038/nri1900 16932750

[10] Ikushima H Negishi H Taniguchi T. The IRF family transcription factors at the interface of innate and adaptive immune responses. Cold Spring Harb Symp Quant Biol (2013) 78:105–16. doi: 10.1101/sqb.2013.78.020321 24092468

[11] Au WC Moore PA Lafleur DW Tombal B Pitha PM. Characterization of the interferon regulatory factor-7 and its potential role in the transcription activation of interferon a genes. J Biol Chem (1998) 273(44):29210–7. doi: 10.1074/jbc.273.44.29210 9786932

[12] Li T Niu X Zhang X Wang S Liu Z. Recombinant human IFNalpha-2b response promotes vaginal epithelial cells defense against candida albicans. Front Microbiol (2017) 8:697. doi: 10.3389/fmicb.2017.00697 28473823 PMC5397410

[13] Stawowczyk M Naseem S Montoya V Baker DP Konopka J Nc R. Pathogenic effects of IFIT2 and interferon-β during fatal systemic candida albicans infection. mBio (2018) 9(2):e00365–18. doi: 10.1128/mBio.00365-18 PMC5904408 29666281

[14] Zhang L Js P. IRF-7, a new interferon regulatory factor associated with Epstein-Barr virus latency. Mol Cell Biol (1997) 17(10):5748–57. doi: 10.1128/MCB.17.10.5748 PMC232423 9315633

[15] Zhang L Js P. Structure and function of IRF-7. J Interferon Cytokine Res (2002) 22(1):95–101. doi: 10.1089/107999002753452700 11846980

[16] Pan H Yan BS Rojas M Shebzukhov YV Zhou H Kobzik L. Ipr1 gene mediates innate immunity to tuberculosis. Nature (2005) 434(7034):767–72. doi: 10.1038/nature03419 PMC1388092 15815631

[17] Honda K Takaoka A Taniguchi T. Type I interferon [corrected] gene induction by the interferon regulatory factor family of transcription factors. Immunity (2006) 25(3):349–60. doi: 10.1016/j.immuni.2006.08.009 16979567

[18] Elhai M Meune C Avouac J Kahan A Allanore Y. Trends in mortality in patients with systemic sclerosis over 40 years: a systematic review and meta-analysis of cohort studies. Rheumatol (Oxford) (2012) 51(6):1017–26. doi: 10.1093/rheumatology/ker269 21900368

[19] Wu M Skaug B Bi X Mills T Salazar G Zhou X. Interferon regulatory factor 7 (IRF7) represents a link between inflammation and fibrosis in the pathogenesis of systemic sclerosis. Ann Rheum Dis (2019) 78(11):1583–91. doi: 10.1136/annrheumdis-2019-215208 PMC7167109 31439591

[20] Smith SG Chen R Kjarsgaard M Huang C Oliveria JP O'byrne PM. Increased numbers of activated group 2 innate lymphoid cells in the airways of patients with severe asthma and persistent airway eosinophilia. J Allergy Clin Immunol (2016) 137(1):75–86.e8. doi: 10.1016/j.jaci.2015.05.037 26194544

[21] He J Yang Q Xiao Q Lei A Li X Zhou P. IRF-7 is a critical regulator of type 2 innate lymphoid cells in allergic airway inflammation. Cell Rep (2019) 29(9):2718–30.e6. doi: 10.1016/j.celrep.2019.10.077 31775040

[22] Li Y Wang C Wu X Tian H Jiang S Xu T. IRF3 and IRF7 contribute to diesel exhaust particles-induced pulmonary inflammation by mediating mTORC1 activation and restraining autophagy in mice. Eur J Immunol (2020) 50(8):1142–53. doi: 10.1002/eji.201948415 32135578

[23] Vonk-Noordegraaf A Haddad F Chin KM Forfia PR Kawut SM Lumens J. Right heart adaptation to pulmonary arterial hypertension: physiology and pathobiology. J Am Coll Cardiol (2013) 62(25 Suppl):D22–33. doi: 10.1016/j.jacc.2013.10.027 24355638

[24] Gurtu V Michelakis ED. Emerging therapies and future directions in pulmonary arterial hypertension. Can J Cardiol (2015) 31(4):489–501. doi: 10.1016/j.cjca.2015.01.028 25840098

[25] Deng Y Guo SL Li JQ Xie SS Zhou YC Wei B. Interferon regulatory factor 7 inhibits rat vascular smooth muscle cell proliferation and inflammation in monocrotaline-induced pulmonary hypertension. Life Sci (2021) 264:118709. doi: 10.1016/j.lfs.2020.118709 33152351

[26] Medzhitov R Horng T. Transcriptional control of the inflammatory response. Nat Rev Immunol (2009) 9(10):692–703. doi: 10.1038/nri2634 19859064

[27] Zhao Y Chen W Zhu W Meng H Chen J Zhang J. Overexpression of interferon regulatory factor 7 (IRF7) reduces bone metastasis of prostate cancer cells in mice. Oncol Res (2017) 25(4):511–22. doi: 10.3727/096504016X14756226781802 PMC7841009 27733217

[28] Xing Y Chen H Guo Z Zhou X. Circular RNA circ0007360 attenuates gastric cancer progression by altering the miR-762/IRF7 axis. Front Cell Dev Biol (2022) 10:789073. doi: 10.3389/fcell.2022.789073 35252169 PMC8891931

[29] Li Y Huang R Wang L Hao J Zhang Q Ling R. microRNA-762 promotes breast cancer cell proliferation and invasion by targeting IRF7 expression. Cell Prolif (2015) 48(6):643–9. doi: 10.1111/cpr.12223 PMC6496530 26597380

[30] Yang Q Li X Chen H Cao Y Xiao Q He Y. IRF7 regulates the development of granulocytic myeloid-derived suppressor cells through S100A9 transrepression in cancer. Oncogene (2017) 36(21):2969–80. doi: 10.1038/onc.2016.448 28092673

[31] Kim JK Jin X Ham SW Lee SY Seo S Kim SC. IRF7 promotes glioma cell invasion by inhibiting AGO2 expression. Tumour Biol (2015) 36(7):5561–9. doi: 10.1007/s13277-015-3226-4 25680411

[32] Tanaka T Murakami K Bando Y. Interferon regulatory factor 7 participates in the M1-like microglial polarization switch. Glia (2015) 63(4):595–610. doi: 10.1002/glia.22770 25422089

[33] Li Z Huang Q Chen H Lin Z Zhao M Jiang Z. Interferon regulatory factor 7 promoted glioblastoma progression and stemness by modulating IL-6 expression in microglia. J Cancer (2017) 8(2):207–19. doi: 10.7150/jca.16415 PMC5327370 28243325

[34] Cohen M Matcovitch O David E Barnett-Itzhaki Z Keren-Shaul H Blecher-Gonen R. Chronic exposure to TGFbeta1 regulates myeloid cell inflammatory response in an IRF7-dependent manner. EMBO J (2014) 33(24):2906–21. doi: 10.15252/embj.201489293 PMC4282639 25385836

[35] Tu D Dou J Wang M Zhuang H Zhang X. M2 macrophages contribute to cell proliferation and migration of breast cancer. Cell Biol Int (2021) 45(4):831–8. doi: 10.1002/cbin.11528 33325089

[36] Tang XD Zhang DD Jia L Ji W Zhao YS. lncRNA AFAP1-AS1 promotes migration and invasion of non-small cell lung cancer via up-regulating IRF7 and the RIG-I-Like receptor signaling pathway. Cell Physiol Biochem (2018) 50(1):179–95. doi: 10.1159/000493967 30278439

[37] Lin L Cai J. Circular RNA circ-EGLN3 promotes renal cell carcinoma proliferation and aggressiveness via miR-1299-mediated IRF7 activation. J Cell Biochem (2020) 121(11):4377–85. doi: 10.1002/jcb.29620 31904147

[38] Yue L Xie Z Li H. Protein tyrosine phosphatase-1B negatively impacts host defense against pseudomonas aeruginosa infection. Am J Pathol (2016) 186(5):1234–44. doi: 10.1016/j.ajpath.2016.01.005 27105736

[39] Leisching G Pietersen RD Van Heerden C Van Helden P Wiid I Baker B. RNAseq reveals hypervirulence-specific host responses to m. tuberculosis infection. Virulence (2017) 8(6):848–58. doi: 10.1128/JVI.00918-08 PMC5626229 27763806

[40] Ruangkiattikul N Nerlich A Abdissa K Lienenklaus S Suwandi A Janze N. cGAS-STING-TBK1-IRF3/7 induced interferon-beta contributes to the clearing of non tuberculous mycobacterial infection in mice. Virulence (2017) 8(7):1303–15. doi: 10.1080/21505594.2017.1321191 PMC5711412 28422568

[41] Cheng Y Schorey JS. Mycobacterium tuberculosis-induced IFN-beta production requires cytosolic DNA and RNA sensing pathways. J Exp Med (2018) 215(11):2919–35. doi: 10.1084/jem.20180508 PMC6219742 30337468

[42] Mathy NW Deng S Gong AY Li M Wang Y Burleigh O. The long non-coding RNA nostrill regulates transcription of Irf7 through interaction with NF-kappaB p65 to enhance intestinal epithelial defense against cryptosporidium parvum. Front Immunol (2022) 13:863957. doi: 10.3389/fimmu.2022.863957 35464447 PMC9021721

[43] Edwards CL Best SE Gun SY Claser C James KR De Oca MM. Spatiotemporal requirements for IRF7 in mediating type I IFN-dependent susceptibility to blood-stage plasmodium infection. Eur J Immunol (2015) 45(1):130–41. doi: 10.1002/eji.201444824 25319247

[44] Bhalla N Gardner CL Downs SN Dunn M Sun C Klimstra WB. Macromolecular synthesis shutoff resistance by myeloid cells is critical to IRF7-dependent systemic interferon Alpha/Beta induction after alphavirus infection. J Virol (2019) 93(24). doi: 10.1128/JVI.00872-19 PMC6880179 31578290

[45] Daffis S Samuel MA Suthar MS Keller BC Gale M Jr Diamond MS. Interferon regulatory factor IRF-7 induces the antiviral alpha interferon response and protects against lethal West Nile virus infection. J Virol (2008) 82(17):8465–75. doi: 10.1128/JVI.00918-08 PMC2519659 18562536

[46] Girkin J Hatchwell L Foster P Johnston SL Bartlett N Collison A. CCL7 and IRF-7 mediate hallmark inflammatory and IFN responses following rhinovirus 1B infection. J Immunol (2015) 194(10):4924–30. doi: 10.4049/jimmunol.1401362 PMC4417644 25847975

[47] Yan W Wei J Deng X. Transcriptional analysis of immune-related gene expression in p53-deficient mice with increased susceptibility to influenza a virus infection. BMC Med Genomics (2015) 8:52. doi: 10.1186/s12920-015-0127-8 26282854 PMC4539693

[48] Rollenhagen C Macura SL Lathrop MJ Mackenzie TA Doncel GF Asin SN. Enhancing interferon regulatory factor 7 mediated antiviral responses and decreasing nuclear factor kappa b expression limit HIV-1 replication in cervical tissues. PLoS One (2015) 10(6):e0131919. doi: 10.1016/j.virol.2018.01.028 26121689 PMC4485897

[49] Ai Z. Revealing key regulators of neutrophil function during inflammation by re-analysing single-cell RNA-seq. PLoS One (2022) 17(10):e0276460. doi: 10.1371/journal.pone.0276460 36269754 PMC9586406

[50] Campbell TM Liu Z Zhang Q Moncada-Velez M Covill LE Zhang P. Respiratory viral infections in otherwise healthy humans with inherited IRF7 deficiency. J Exp Med (2022) 219(7). doi: 10.1084/jem.2022020210282022c PMC9178406 35670811

[51] Ke J Ca A Cn J Lange Pt VlT. Interferon regulatory factor 7 attenuates chronic gammaherpesvirus infection. J Virol (2020) 94(24):e01554–20. doi: 10.1016/j.ajpath.2016.01.005 PMC7925175 32967960

[52] Zhou S Cerny AM Fitzgerald KA Kurt-Jones EA Finberg RW. Role of interferon regulatory factor 7 in T cell responses during acute lymphocytic choriomeningitis virus infection. J Virol (2012) 86(20):11254–65. doi: 10.1128/JVI.00576-12 PMC3457134 22875973

[53] Li W Hofer MJ Nocon AL Manders P Campbell IL. Interferon regulatory factor 7 (IRF7) is required for the optimal initial control but not subsequent clearance of lymphocytic choriomeningitis virus infection in mice. Virology (2013) 439(2):152–62. doi: 10.1016/j.virol.2013.02.015 23490048

[54] Xue Q Liu H Zhu Z Yang F Ma L Cai X. Seneca Valley virus 3C(pro) abrogates the IRF3- and IRF7-mediated innate immune response by degrading IRF3 and IRF7. Virology (2018) 518:1–7. doi: 10.1016/j.virol.2018.01.028 29427864

[55] Yang K Xue Y Niu T. African Swine fever virus MGF505-7R protein interacted with IRF7and TBK1 to inhibit type I interferon production. Virus Res (2022) 322:198931. doi: 10.1016/j.virusres.2022.198931 36130654

[56] Gao L Li K Zhang Y Liu Y Liu C Zhang Y. Inhibition of DNA-sensing pathway by marek's disease virus VP23 protein through suppression of interferon regulatory factor 7 activation. J Virol (2019) 93(4):e01934-18. doi: 10.1128/JVI.01934-18 30518647 PMC6363996

[57] Yang F Xu Z Duan S. MicroRNA-541 promotes the proliferation of vascular smooth muscle cells by targeting IRF7. Am J Transl Res (2016) 8(2):506–15. PMC4846900 27158343

[58] Lopez CB Rosenberger CM Podyminogin RL Diercks AH Treuting PM Peschon JJ. miR-144 attenuates the host response to influenza virus by targeting the TRAF6-IRF7 signaling axis. PLoS Pathog (2017) 13(4):e1006305. doi: 10.1371/journal.ppat.1006305 28380049 PMC5393898

[59] Unutmaz D Higgs R Lazzari E Wynne C Ní Gabhann J Espinosa A. Self protection from anti-viral responses – Ro52 promotes degradation of the transcription factor IRF7 downstream of the viral toll-like receptors. PLoS One (2010) 5(7):e11776. doi: 10.1371/journal.pone.0011776 20668674 PMC2909902

[60] Yu Y Hayward GS. The ubiquitin E3 ligase RAUL negatively regulates type I interferon through ubiquitination of the transcription factors IRF7 and IRF3. Immunity (2010) 33(6):863–77. doi: 10.1016/j.immuni.2010.11.027 PMC3012379 21167755

